# Supplementation with a fish protein hydrolysate (*Micromesistius poutassou*): effects on body weight, body composition, and CCK/GLP-1 secretion

**DOI:** 10.3402/fnr.v60.29857

**Published:** 2016-01-29

**Authors:** Vincenzo Nobile, Elisa Duclos, Angela Michelotti, Gioia Bizzaro, Massimo Negro, Florian Soisson

**Affiliations:** 1 Farcoderm Srl member of Complife Group, Pavia, Italy; 2 Compagnie des Pêches Saint-Malo Santé, Saint Malo, France; 3 Laboratory of Pharmacobiochemistry, Sports Nutrition and Nutriceuticals, University of Pavia, Pavia, Italy

**Keywords:** blue whiting, fish protein hydrolysate, body weight, cholecystokinin, glucagon-like peptide-1, body composition, clinical study, weight management

## Abstract

**Background:**

Fish protein hydrolysates (FPHs) have been reported as a suitable source of proteins for human nutrition because of their balanced amino acid composition and positive effect on gastrointestinal absorption.

**Objective:**

Here, we investigated the effect of a FPH, Slimpro^®^, obtained from blue whiting (*Micromesistius poutassou*) muscle by enzymatic hydrolysis, on body composition and on stimulating cholecystokinin (CCK) and glucagon-like peptide-1 (GLP-1) secretion.

**Design:**

A randomized clinical study was carried out on 120, slightly overweight (25 kg/m^2^ ≤ BMI<30 kg/m^2^), male (25%) and female (75%) subjects. FPH was tested in a food supplement at two doses (1.4 and 2.8 g) to establish if a dose–effect relationship exists. Product use was associated with a mild hypocaloric diet (−300 kcal/day). Body composition (body weight; fat mass; extracellular water; and circumference of waist, thighs, and hips) and CCK/GLP-1 blood levels were measured at the beginning of the study and after 45 and 90 days of product use. CCK/GLP-1 levels were measured since they are involved in controlling food intake.

**Results:**

Treated subjects reported an improvement of body weight composition and an increased blood concentration of both CCK and GLP-1. No differences were found between the 1.4 and 2.8 g FPH doses, indicating a plateau effect starting from 1.4 g FPH.

**Conclusions:**

Both 1.4 and 2.8 g of FPH were effective in improving body composition and in increasing CCK and GLP-1 blood levels.

Peptides derived from hydrolyzed food proteins are of big interest for nutritional and pharmaceutical application. Fish protein hydrolysates (FPHs) have been reported as a suitable source of proteins for human nutrition because of their balanced amino acid composition and positive effect on gastrointestinal absorption (1). Recent studies demonstrated that FPHs have a number of physiological effects beyond their role as source of amino acids and energy (2). Studies on fish-derived peptides demonstrated antihypertensive (3–6), antioxidant (7–10), immunomodulating effects (11), reparative properties in the intestine (12, 13), and effects in reducing plasma cholesterol and triglycerides levels (2, 14–17).

Energy balance is regulated by both short-term and long-term negative feedback. In the short term, meal size is regulated by various ‘satiation signals’, such as the secretion of the peptide cholecystokinin (CCK) from duodenal I cells and glucagon-like peptide-1 (GLP-1) from intestinal L cells in response to nutrient ingestion. Nonetheless, peripheral GLP-1 may also interact with known regulators of long-term energy balance such as leptin, implicating it in both rapid and long-term regulation of energy balance. Whereas reports on the above relationship between peripheral GLP-1 and leptin are relatively new, evidence of a relationship between central GLP-1 and leptin dates back to 1997, when Goldstone and colleagues reported the presence of leptin receptors on mouse hindbrain proglucagon gene (GCG) expressing neurons, as well as the blockade of intracerebroventricular-leptin-induced anorexia in rats by intracerebroventricular exendin 9 (18). Further support for this hypothesis has come from the findings that rat hindbrain *GCG* neurons are activated by leptin. Moreover, leptin directly depolarizes mouse hindbrain *GCG* neurons (19, 20).

Even if CCK and GLP-1 are directly involved in the short-term food intake control, the absence of CCK and GLP-1 receptors, in mice, seems not to be essential in the long-term maintenance of body weight (21–23). In contrast to the study of Kopin et al. (21), Kawano et al. (24) reported body weight increase in Otsuka Long-Evans Tokushima Fatty (OLETF) rats lacking CCK-A receptor. The apparent discrepancy between OLETF and CCK-AR^−/−^ may be explained by interspecies differences (e.g. tissue distribution of receptors) influencing the physiological alterations due to CCK-AR absence. Additionally, co-morbidity in transgenic/selected mice strains could partly explain the controversial data. These findings in mice cannot exclude that CCK and GLP-1 are not essential for the long-term maintenance of body weight in other species with a genetic background and/or compensatory mechanisms that are considerably different from mice. Further studies on humans are needed to unravel the complexity of CCK/GLP-1 effects in body weight maintenance.

Alteration in energy balance leads to overweight/obesity due to a negative balance between energy intake and expenditure. The incidence of overweight and obesity has increased dramatically worldwide. Recent data indicate that there are more than 1.9 billion overweight people worldwide, representing about 39% of the adult population (25). Modulation of physiological pathways capable of suppressing appetite and thereby reducing energy intake provides an interesting approach to develop weight management strategies. In this context, molecules capable of stimulating the secretion of both CCK and GLP-1 provide a logical and natural approach to achieve this aim.

Previous studies demonstrated the efficacy of a FPH obtained from from blue whiting (*Micromesistius poutassou*) muscle on STC-1 (secretin tumor cell) cell line (26), rats (27), and humans (28) in stimulating the release of CCK and GLP-1. Based on these preliminary data, we conducted a clinical study in humans to confirm the weight management efficacy of FPH. To our knowledge, no other studies have investigated the long-term effects of FPH in stimulating CCK and GLP-1 secretion and therefore in controlling body weight.

## Experimental section

### Study design

This monocentric, randomized study was carried out in accordance with the Declaration of Helsinki and the Good Clinical Practice guidelines E6 (R1). The study protocol and the informed consent form were approved by the ‘Independent Ethical Committee for Non-Pharmacological Clinical trials’ during its meeting on December 17, 2013. All subjects provided written informed consent before initiation of any study-related procedures. The study took place at Farcoderm Srl facilities in San Martino Siccomario (PV), Italy. Farcoderm Srl is an independent testing laboratory for *in vitro* and *in vivo* safety and efficacy assessment of cosmetics, food supplements, and medical devices.

### Subjects

Eligible subjects were all adult, slightly overweight (25 kg/m^2^ ≤BMI< 30 kg/m^2^), male (25%) and female (75%) subjects aged between 18 and 55 years. Subjects were of general good health, had no alimentary/eating disorders (i.e. no bulimia or psychogenic eating disorders, etc.), and known history of metabolic syndrome. Exclusion criteria were pregnancy or intention to become pregnant, lactation, pre-menopause/menopause, food intolerances/allergy, pharmacological treatments known to interfere with the tested product or having an effect on metabolism, participation in another similar study (at least 6 months prior to enrolling in the study), and unwillingness or inability to comply with the requirements of the study protocol. The study further excluded subjects using food supplements containing ingredients having influence on body weight.

### Alimentary habits screening

Two weeks before the study started subjects were asked to fill in an alimentary diary reporting their food preferences. A mild hypocaloric (−300 kcal/day) diet was elaborated for each subject by a dietitian based on subject's food preferences and habits as reported in the alimentary diary. Approximately, 55% of energy intake was from carbohydrates, 25% from lipids, and the remaining 20% from proteins.

### Interventions

The tested product was a commercially available food supplement containing Slimpro^®^ (Compagnie des Pêches Saint-Malo Santé, Saint-Malo, France). Slimpro content in the food supplement (per 25 g, one dose) was 1.4 g. Both the active (one dose treatment arm) and the placebo products were taken as follows: ‘dilute the content of one sachet in a large glass of cool water (200 ml). Shake or stir with a spoon. Consume within 10–30 min before the main meal’. In the case of two-dose treatment arm, one sachet of the active product was taken 30 min before lunch and one sachet 30 min before dinner. In the placebo product, Slimpro was replaced by whey protein isolate.

### Endpoints

**Fig. 1:**
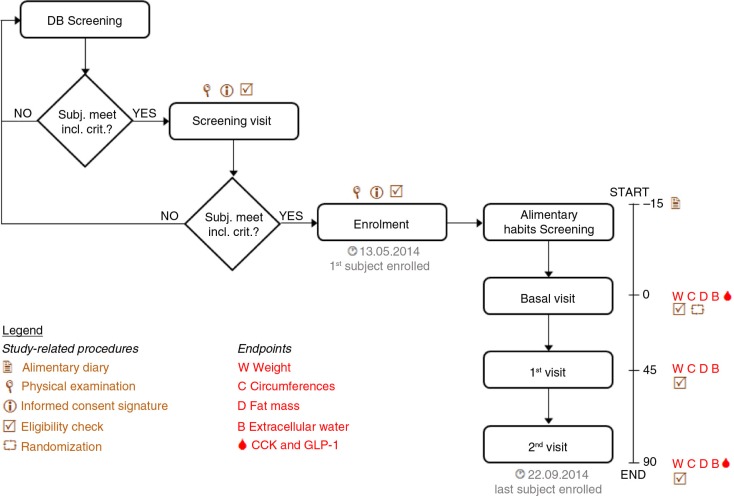
Study flow and schedule of assessments chart. Screening of eligible trial participants in the Farcoderm volunteers database using the keywords: Sex = “female” and “male”, “18” < Age < “55” for males and “18” < Age < “45” for females, Menopause: “No”, Testing preferences: “Dietary supplements”.

Primary efficacy endpoints were body weight, fat mass, and safety of use. Secondary efficacy endpoints were extracellular water; circumference of waist, hips, and thighs; CCK; and GLP-1 levels in blood. The study flow and the schedule of assessments chart are reported in Fig. 1.

### Body composition measurements

Body weight and height were measured under standardized anthropometric operative procedures using an electronic balance (model PP3020, Tefal, France) and an upright stadiometer (model 27328, Gima, Italy). Both body weight and height were measured with the subject barefoot and wearing underwear.

Fat mass was measured using Dual Energy X-ray Absorptiometry (Lunar Prodigy Primo™, GE Healthcare).

Extracellular water was measured in the supine position, according to NIH consensus statement (29), using BIA technique (Model BIA 101 Anniversary ASE, Akern, Italy). BIA data were analyzed using the manufacturer's software Bodygram PLUS (version 1.0) running on Windows 8.1 Pro (Microsoft, USA).

Circumference of waist, hips, and thighs (left and right) were measured using a flexible measuring tape (precision: 1 mm). Measures were taken according to NHANES III guidelines (30), as follows: 1) circumference of waist was measured extending the tape around the waist above the right/left iliac crest; the measurement was taken at minimal inspiration, 2) the circumference of thighs (left and right) was measured extending the tape around the right and left thigh, in the area of maximum extension (approximately mid-thigh); to improve measurement reproducibility from visit to visit, the distance from the iliac crest was measured, and 3) the circumference of hips was measured extending the tape at the maximum extension of the hips.

### Biochemical analysis

Total CCK and GLP-1 levels in fasting blood plasma were assessed using commercially available ELISA (enzyme-linked immunosorbent assay) kits according to the manufacturer's instructions. Human (CCK) ELISA kit (catalogue no. 201-12-1358) by SunRed was used for CCK measurement. Human (GLP-1) ELISA kit (catalogue no. 201-12-0023) by SunRed was used for GLP-1 measurement. Detection limits were 0.4 ng/L for the CCK ELISA kit and 0.6 pmol/L for the GLP-1 ELISA kit. Intra- and interassay coefficient of variation (CV) for CCK ELISA kit were <10% and <12%, respectively; while no CV was available for the GLP-1 ELISA kit.

### Randomization

Subjects were assigned to treatment groups using a computer-generated restricted randomization list “Wey’s urn” (algorithm), using PASS 11 statistical software (version 11.0.8 for Windows; PASS, Kaysville, UT, USA). Subjects were randomized in a 1:1:1 (1.4 g/2.8 g/placebo) ratio. The software was running on Windows Server 2008 R2 Standard SP1 64 bit Edition (Microsoft, USA). Subjects, investigator and collaborators were kept blind to products assignment. Sequentially numbered, opaque, and sealed envelopes, reporting the unblinded treatment allocation (based on subject randomization number), were prepared for each subject and stored in a safe place by the *in site* study director.

### Sample size

The number of subjects was calculated based on a reduction in baseline body weight after 3 months ≥1.3% using a two-sided test of significance at the 5% level and 80% power. Sample size was calculated using PASS 11 statistical software (version 11.0.8 for Windows) running on Windows Server 2008 R2 Standard SP1 64 bit edition (Microsoft, USA). A sample size of 20 subjects per group was necessary, given an anticipate dropout rate by 20%.

### Statistical methods

Statistical analysis was performed using NCSS 8 (version 8.0.4 for Windows; NCSS, kaysville, UT, USA) running on Windows Server 2008 R2 Standard SP1 64 bit edition (Microsoft, USA). Data normality was checked using Shapiro–Wilk *W* normality test and data shape. Intragroup and intergroup comparisons were carried out using repeated measures analysis of variance (RM-ANOVA) followed by Tukey–Kramer post-test. A p-value <0.05 was considered statistically significant. Intragroup statistical analysis was carried out on raw data, whereas intergroup statistical analysis was carried out on the changes from baseline.

## Results

### Subjects

**Fig. 2:**
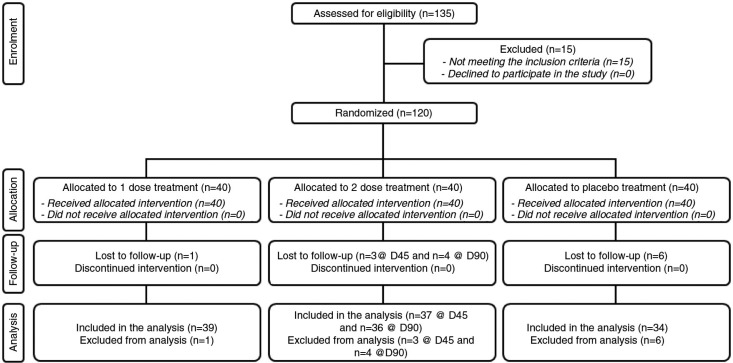
Flow chart of inclusion of subjects.

The study was conducted between May and September 2014. A total of 30 male and 90 female subjects were successfully randomized (Fig. 2). Eleven subjects (9.2%) were lost to follow-up. The population was Caucasian. Demographic and baseline characteristics (Table 1) were similar across treatment arms, indicating an unbiased randomization and the absence of covariates. One subject in the one-dose treatment arm, four subjects in the two-dose treatment arm, and six subjects in the placebo treatment arm discontinued intervention because they were no longer interested to participate in the study. The per-protocol population consisted of 109 subjects. All subjects were included in the safety analysis data set. All the tested products were well tolerated. No adverse reactions occurred during the study period.

**Table 1:** Demographic and baseline characteristics

	One dose	Two doses	Placebo
Sex			
Male,% (n)	25 (10)	25 (10)	25 (10)
Female,% (n)	75 (30)	75 (30)	75 (30)
Age (years)	41.3±1.2	39.1±1.2	41.1±1.3
Height (m)	1.67±0.01	1.66±0.01	1.66±0.01
Weight (kg)	79.5±1.5	77.9±1.7	76.0±1.6
Body mass index (kg/m^2^)	28.4±0.2	28.1±0.3	27.5±0.3
Fat mass (kg)	24.7±0.6	24.6±0.5	23.6±0.5
Extracellular water (L)	18.0±0.5	17.4±0.4	17.8±0.5
Circumference of waist (cm)	90.0±1.0	89.4±1.1	88.8±1.2
Circumference of hips (cm)	111.6±1.3	109.0±1.2	110.3±1.2
Circumference of thighs (cm)	57.6±0.7	57.9±0.7	56.8±0.6
CCK (ng/L)	16.6±1.4	17.3±1.2	17.4±1.5
GLP-1 (pmol/L)	26.5±2.3	26.8±2.1	25.8±2.1

Data are means±SE. CCK, cholecystokinin; GLP-1, glucagon-like peptide-1.

### Body composition measurement

**Fig. 3:**
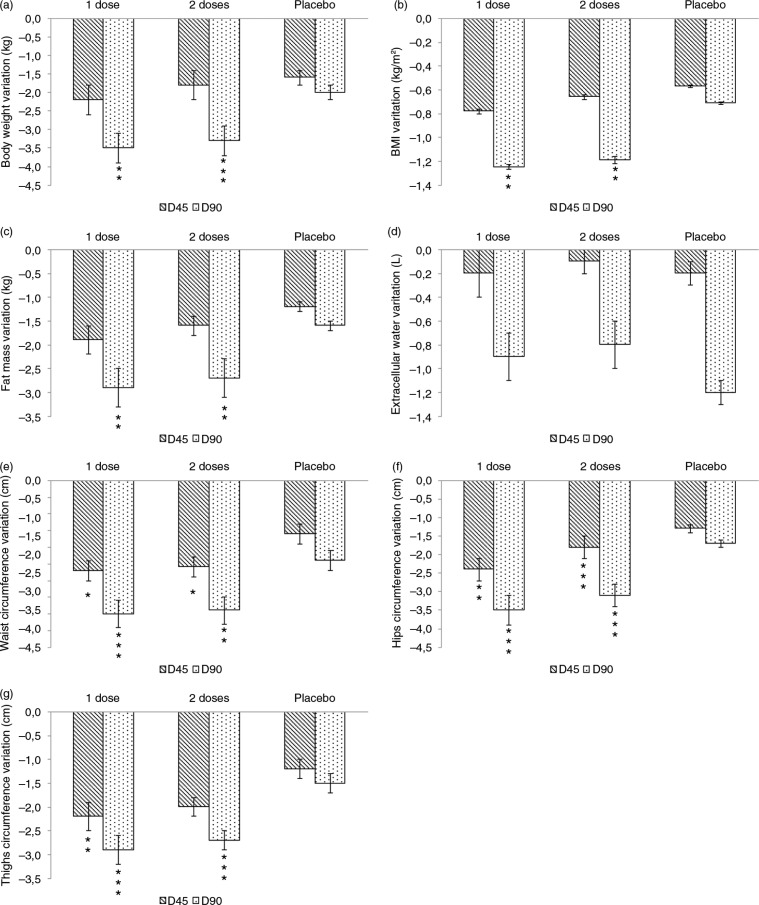
Body composition, body weight, and BMI. (a) Body weight, (b) body mass index, (c) fat mass, (d) extracellular water, (e) circumference of waist, (f) circumference of hips, and (g) circumference of thighs. Statistical analysis (vs. placebo) is reported as follows: *p<0.05, **p<0.01, and ***p<0.001. Values are expressed as differences from baseline and are given as mean±SE.

Data on body composition are reported in Table 2 and in Fig. 3. Baseline levels for all the measured parameters were similar and not statistically significant (p>0.05) for each treatment arm.

**Table 2:** Body composition

		One dose (1.4 FPH g)	Two doses (2.8 FPH g)	Placebo
Body weight (kg)	D0	79.5±1.5	77.9±1.7	76.0±1.6
	D45	77.3±1.4 (−2.2)***	75.7±1.7 (−1.8)***	73.9±1.8 (−1.6)***
	D90	76.0±1.4 (−3.5)***	74.3±1.7 (−3.3)***	73.5±1.8 (−2.0)***
BMI (kg/m^2^)	D0	28.4±0.2	28.1±0.3	27.5±0.3
	D45	27.6±0.3 (−0.8)***	27.5±0.3 (−0.7)***	26.9±0.3 (−0.6)***
	D90	27.1±0.3 (−1.3)***	27.0±0.3 (−1.2)***	26.8±0.3 (−0.7)***
Fat mass (kg)	D0	24.7±0.6	24.6±0.5	23.6±0.5
	D45	22.9±0.6 (−1.9)***	22.8±0.6 (−1.6)***	22.5±0.6 (−1.2)***
	D90	21.7±0.7 (−2.9)***	21.7±0.6 (−2.7)***	22.1±0.6 (−1.6)***
Extracellular water (L)	D0	18.0±0.5	17.4±0.4	17.8±0.5
	D45	17.8±0.4 (−0.2)	17.2±0.4 (−0.1)	17.5±0.5 (−0.2)
	D90	17.2±0.5 (−0.9)***	16.5±0.4 (−0.8)***	16.5±0.5 (−1.2)***
Circumference of waist (cm)	D0	90.0±1.0	89.4±1.1	88.8±1.2
	D45	87.3±1.0 (−2.7)***	87.0±1.1 (−2.6)***	87.2±1.4 (−1.6)***
	D90	85.9±1.0 (−4.0)***	85.9±1.1 (−3.9)***	86.5±1.3 (−2.4)***
Circumference of thighs (cm)	D0	57.6±0.7	57.9±0.7	56.8±0.6
	D45	55.5±0.6 (−2.2)***	55.9±0.7 (−2.0)***	55.5±0.6 (−1.2)***
	D90	54.6±0.6 (−2.9)***	55.0±0.6 (−2.7)***	55.1±0.6 (−1.5)***
Circumference of hips (cm)	D0	111.6±1.3	109.0±1.2	110.3±1.2
	D45	109.2±1.3 (−2.4)***	107.2±1.2 (−1.8)***	109.2±1.3 (−1.3)***
	D90	108.3±1.3 (−3.5)***	105.5±1.2 (−3.1)***	108.8±1.3 (−1.7)***

Intragroup statistical analysis is reported as follows: *p;<0.05, **p<0.01, and ***p<0.001. Data are mean±SE. Changes from baseline are given in brackets. FPH, fish protein hydrolysate.

Weight was decreased both for the 1.4 and 2.8 g FPH doses. As expected, weight was decreased also in the placebo treatment arm. Differences between the 1.4 and 2.8 g doses and placebo after 90 days of treatment were statistically significant (p<0.001 and <0.01 for the 1.4 and 2.8 g dose, respectively, Fig. 3).

BMI was decreased both for the 1.4 and 2.8 g FPH doses. As expected, BMI was decreased also in the placebo treatment arm. Differences between the 1.4 and 2.8 g dose and placebo after 90 days of treatment were statistically significant (p<0.01, Fig. 3).

Fat mass was decreased both for the 1.4 g and 2.8 g FPH doses. As expected, fat mass was decreased also in the placebo treatment arm. Differences between the 1.4 and 2.8 g dose and placebo after 90 days of treatment were statistically significant (p<0.01, Fig. 3).

Extracellular water was decreased both for the 1.4 and 2.8 g FPH doses after 90 days of treatment. In the placebo treatment arm, extracellular water was decreased after 90 days of treatment. Differences between the 1.4 and 2.8 g doses and placebo were not statistically significant (p>0.05, Fig. 3).

Waist circumference was decreased both for the 1.4 and 2.8 g FPH doses. As expected, waist circumference was decreased also in the placebo treatment arm. Differences between the 1.4 and 2.8 g dose and placebo were statistically significant (p < 0.05 both for the 1.4 and 2.8 g dose after 45 days of treatment, p < 0.001, for the 1.4 g dose after 90 days of treatment, and p < 0.01 for the 2.8 g dose after 90 days of treatment, Fig. 3).

The circumference of thighs was decreased both for the 1.4 and 2.8 g FPH doses. As expected, the circumference of thighs was decreased also in the placebo treatment arm. Differences between the 1.4 and 2.8 g dose and placebo were statistically significant (p<0.01 for the 1.4 g dose after 45 days of treatment, p<0.001 for the 2.8 g dose after 45 days of treatment, and p<0.001 both for the 1.4 and 2.8 g dose after 90 days of treatment, Fig. 3).

Hip circumference was decreased both for the 1.4 and 2.8 g FPH doses. As expected, hip circumference was decreased also in the placebo treatment arm. Differences between the 1.4 and 2.8 g dose and placebo were statistically significant (p<0.01 for the 1.4 g concentration after 45 days of treatment and p<0.001 for both the 1.4 and 2.8 g dose after 90 days of treatment, Fig. 3).

Differences between the 1.4 and 2.8 g FPH dose for all the measured endpoints were not statistically significant.

### CCK and GLP-1 blood levels

**Fig. 4:**
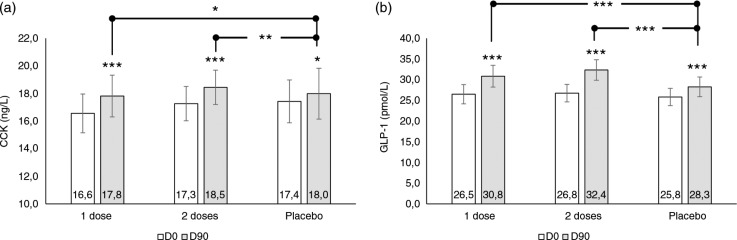
Blood biomarker levels. (a) CCK blood levels and (b) GLP-1 blood levels. Intragroup (vs. D0) statistical analysis is reported upon the bars of the histogram. The lines report the intergroup (vs. placebo) statistical analysis. Statistical analysis is reported as follows: *p<0.05, **p<0.01, and ***p<0.001. Data are mean±SE.

Data on CCK and GLP-1 blood levels are reported in Fig. 4. Baseline levels for both CCK and GLP-1 were similar and not statistically significant (p>0.05) for each treatment arm.

CCK blood levels were increased both for the 1.4 and 2.8 g doses. Differences between the 1.4 and 2.8 g dose and placebo were statistically significant (p<0.05 and <0.01 for the 1.4 and 2.8 g doses, respectively, Fig. 4).

GLP-1 blood levels were increased both for the 1.4 and 2.8 g doses. Differences between the 1.4 and 2.8 g dose and placebo were statistically significant (p<0.001, Fig. 4). In the placebo treatment arm, an increase of both CCK and GLP-1 was observed.

## Discussion

The effects of CCK and GLP-1 in controlling food intake in the short time are well known. However, controversial data were found in animal models regarding their involvement in weight maintenance (21–24). On the other hand, no studies were found in the literature about the long-term effects of CCK and GLP-1 secretion on body composition. The aim of our study was to investigate the effect of a FPH, obtained from blue whiting (*M. poutassou*) muscle by enzymatic hydrolysis, on body composition and on CCK and GLP-1 blood levels over a 90-day study period. Two FPH doses (1.4 and 2.8 g) were investigated to choose the best effective dose.

Both the 1.4 and 2.8 g FPH dose increased the serum levels of CCK and GLP-1. The increase of CCK and GLP-1 was statistically significant when compared with placebo without any differences among the two tested doses. The results obtained in this study confirmed the preliminary results obtained in the earlier *in vitro* and *in vivo* studies carried out on blue whiting FPH Slimpro (26–28). As a consequence of FPH consumption, body composition was changed. Body weight, BMI, fat mass, and circumferences (waist, thighs, and hips) were statistically significantly decreased when compared with placebo after 90 days of treatment. Notably, the decrease in body weight was not related to extracellular water decrease (‘draining effect’) but was related to fat mass decrease (‘slimming effect’).

As expected, an improvement of body composition was also seen in the placebo group due to the caloric restriction (−300 kcal) during the study period. CCK/GLP-1 blood concentration was also increased in the placebo group. This finding is consistent with the evidence that suboptimal energy intake, reduced body weight, or both may be associated with increased plasma CCK concentrations (31–33). No clear evidences were found in the literature between caloric restriction and the increase in GLP-1 blood concentration.

No differences in CCK/GLP-1 blood concentrations and changes in markers of body composition were found between the 1.4 and 2.8 g concentration, indicating a plateau effect starting from the 1.4 g FPH doses. The putative mechanism of action of FPH in body weight management could be as follows: 1) an increase of CCK and GLP-1 blood levels, 2) a decrease in food intake (meal size) stimulated by CCK and GLP-1 secretion in the short term, and 3) a decrease of adipose tissue mass mediated by GLP-1 interaction with leptin in the long term. According to the postulated mechanism of action, the decrease in body weight is mediated by two mechanisms: the first one (indirect) is related to the decrease of food intake, leading to a decrease in the total calories; the second one (direct) is related to the decrease of adipose tissue mass. In addition to the stimulation of endogenous CCK, part of the effect could be due to the exogenous CCK contained in the FPH.

Even though a positive correlation between FPH intake, CCK/GLP-1 blood levels increase, and body weight management was demonstrated, further studies are needed to unravel the mechanism of action of the tested product.

## Conclusions

Our results confirm the previous *in vitro* and *in vivo* (26–28) results of the efficacy of a FPH, Slimpro^®^, obtained from blue whiting (*M. poutassou*) muscle by enzymatic hydrolysis in stimulating the secretion of CCK and GLP-1. Moreover, Slimpro was effective on body weight and particularly in controlling body composition. In conclusions, the intake of Slimpro obtained from blue whiting (*M. poutassou*) muscle by enzymatic hydrolysis can be considered a nutrition strategy for body weight control. To the best of our knowledge, this is the first study demonstrating an increase of CCK and GLP-1 correlated to a decrease of body weight.

## References

[1] Benjakul S, Yarnpakdee S, Senphan T, Halldorsdottir SM, Kristinsson HG, Kristinsson HG (2014). Fish protein hydrolysates: production, bioactivities and applications. Antioxidants and functional components in aquatic foods.

[2] Moller NP, Scholz-Ahrens KE, Roos N, Schrezenmeir J (2008). Bioactive peptides and proteins from foods: indication for health effects. Eur J Nutr.

[3] Hatanaka A, Miyahara H, Suzuki KI, Sato S (2009). Isolation and identification of antihypertensive peptides from Antarctic krill tail meat hydrolysate. J Food Sci.

[4] Kim SK, Ngo DH, Vo TS (2012). Marine fish-derived bioactive peptides as potential antihypertensive agents. Adv Food Nutr Res.

[5] Li Y, Zhou J, Huang K, Sun Y, Zeng X (2012). Purification of a novel angiotensin I converting enzyme (ACE) inhibitory peptide with an antihypertensive effect from loach (*Misgurnus anguillicaudatus*). J Agric Food Chem.

[6] Ngo DH, Ryu B, Vo TS, Himaya SW, Wijesekara I, Kim SK (2011). Free radical scavenging and angiotensin-I converting enzyme inhibitory peptides from Pacific cod (*Gadus macrocephalus*) skin gelatin. Int J Biol Macromol.

[7] Nazeer RA, Sampath Kumar NS, Jai Ganesh R (2012). In vitro and in vivo studies on the antioxidant activity of fish peptide isolated from the croaker (*Otolithes ruber*) muscle protein hydrolysate. Peptides.

[8] Najafian L, Babji AS (2012). A review of fish-derived antioxidant and antimicrobial peptides: their production, assessment, and applications. Peptides.

[9] Sampath Kumar NS, Nazeer RA, Jaiganesh R (2012). Purification and identification of antioxidant peptides from the skin protein hydrolysate of two marine fishes, horse mackerel (*Magalaspis cordyla*) and croaker (*Otolithes ruber*). Amino Acids.

[10] Yang R, Wang J, Liu Z, Pei X, Han X, Li Y (2011). Antioxidant effect of a marine oligopeptide preparation from chum salmon (*Oncorhynchus keta*) by enzymatic hydrolysis in radiation injured mice. Mar Drugs.

[11] Duarte J, Vinderola G, Ritz B, Perdigon G, Matar C (2006). Immunomodulating capacity of commercial fish protein hydrolysate for diet supplementation. Immunobiology.

[12] Fitzgerald AJ, Rai PS, Marchbank T, Taylor GW, Ghosh S, Ritz BW (2005). Reparative properties of a commercial fish protein hydrolysate preparation. Gut.

[13] Marchbank T, Limdi JK, Mahmood A, Elia G, Playford RJ (2008). Clinical trial: protective effect of a commercial fish protein hydrolysate against indomethacin (NSAID)-induced small intestinal injury. Aliment Pharmacol Ther.

[14] Rigamonti E, Parolini C, Marchesi M, Diani E, Brambilla S, Sirtori CR (2010). Hypolipidemic effect of dietary pea proteins: impact on genes regulating hepatic lipid metabolism. Mol Nutr Food Res.

[15] Sugiyama K, Ohkawa S, Muramatsu K (1986). Relationship between amino acid composition of diet and plasma cholesterol level in growing rats fed a high cholesterol diet. J Nutr Sci Vitaminol (Tokyo).

[16] Shukla A, Bettzieche A, Hirche F, Brandsch C, Stangl GI, Eder K (2006). Dietary fish protein alters blood lipid concentrations and hepatic genes involved in cholesterol homeostasis in the rat model. Br J Nutr.

[17] Bjørndal B, Berge C, Ramsvik MS, Svardal A, Bohov P, Skorve J (2013). A fish protein hydrolysate alters fatty acid composition in liver and adipose tissue and increases plasma carnitine levels in a mouse model of chronic inflammation. Lipids Health Dis.

[18] Williams DL, Baskin DG, Schwartz MW (2006). Leptin regulation of the anorexic response to glucagon-like peptide-1 receptor stimulation. Diabetes.

[19] Hisadome K, Reimann F, Gribble FM, Trapp S (2010). Leptin directly depolarizes preproglucagon neurons in the nucleus tractus solitarius: electrical properties of glucagon-like peptide 1 neurons. Diabetes.

[20] Elias CF, Kelly JF, Lee CE, Ahima RS, Drucker DJ, Saper CB (2000). Chemical characterization of leptin-activated neurons in the rat brain. J Comp Neurol.

[21] Kopin A, Foulds MW, McBride EW, Nguyen M, Al-Haider W, Schmitz F (1999). The cholecystokinin-A receptor mediates inhibition of food intake yet is not essential for the maintenance of body weight. J Clin Invest.

[22] Scrocchi LA, Brown TJ, MaClusky N, Brubaker PL, Auerbach AB, Joyner AL (1996). Glucose intolerance but normal satiety in mice with a null mutation in the glucagon-like peptide 1 receptor gene. Nat Med.

[23] Erickson JC, Clegg KE, Palmiter RD (1996). Sensitivity to leptin and susceptibility to seizures of mice lacking neuropeptide Y. Nature.

[24] Kawano K, Hirashima T, Mori S, Saitoh Y, Kurosumi M, Natori T (1992). Spontaneous long-term hyperglycemic rat with diabetic complications. Otsuka Long-Evans Tokushima Fatty (OLETF) strain. Diabetes.

[25] World Health Organization (2015). Fact sheet 311 – obesity and overweight.

[26] Cudennec B, Ravallec-Plé R, Courois E, Fouchereau-Peron M (2008). Peptides from fish and crustacean by-products hydrolysates stimulate cholecystokinin release in STC-1 cells. Food Chem.

[27] Cudennec B, Fouchereau-Peron M, Ferry F, Duclos E, Ravallec R (2012). In vitro and in vivo evidence for a satiating effect of fish protein hydrolysate obtained from blue whiting (*Micromesistius poutassou*) muscle. J Func Foods.

[28] Zaïr Y, Duclos E, Housez B, Vergara C, Cazaubiel M, Soisson F (2014). Evaluation of the satiating properties of a fish protein hydrolysate among overweight women: a pilot study. Nutr Food Sci.

[29] NIH Consensus Statement (1996). Bioelectrical impedance analysis in body composition measurement: National Institute of Health Technology Assessment Conference Statement. December 12–14, 1994. Nutrition.

[30] National Health and Nutrition Examination Survey III (1998). Body measurements (anthropometry).

[31] 
Pirke KM, Kellner MB, Friess E, Krieg JC, Fichter MM (1994). Satiety and cholecystokinin. Int J Eat Disord.

[32] Tamai H, Takemura J, Kobayashi N, Matsubayashi S, Matsukura S, Nakagawa T (1993). Changes in plasma cholecystokinin concentrations after oral glucose tolerance test in anorexia nervosa before and after therapy. Metabolism.

[33] Näslund E, Melin I, Grybäck P, Hägg A, Hellström PM, Jacobsson H (1997). Reduced food intake after jejunoileal bypass: a possible association with prolonged gastric emptying and altered gut hormone patterns. Am J Clin Nutr.

